# Low-molecular-weight heparin for prevention of placenta-mediated pregnancy complications: protocol for a systematic review and individual patient data meta-analysis (AFFIRM)

**DOI:** 10.1186/2046-4053-3-69

**Published:** 2014-06-26

**Authors:** Marc A Rodger, Nicole J Langlois, Johanna IP de Vries, Évelyne Rey, Jean-Christophe Gris, Ida Martinelli, Ekkehard Schleussner, Timothy Ramsay, Ranjeeta Mallick, Becky Skidmore, Saskia Middeldorp, Shannon Bates, David Petroff, Dick Bezemer, Marion E van Hoorn, Carolien NH Abheiden, Annalisa Perna, Paulien de Jong, Risto Kaaja

**Affiliations:** 1 The Ottawa Hospital, Centre for Practice-Changing Research, 501 Smyth Road, Box 201, Ottawa, ON K1H 8 L6, Canada; 2 The Ottawa Hospital Research Institute, Centre for Practice-Changing Research, 501 Smyth Road, Box 201, Ottawa, ON K1H 8 L6, Canada; 3 Department of Obstetrics and Gynaecology, VU University Medical Center, PO Box 7057, Amsterdam MB 1007, The Netherlands; 4 CHU Ste-Justine, 3175 chemin de la Côte-Sainte-Catherine, local 4804, Montreal, QC H3T 1C5, Canada; 5 Consultations et Laboratoire d'Hématologie & Délégation à la Recherche Clinique et à l'Innovation, Place du Pr. Robert Debré, Nîmes cédex 09 F-30029, France; 6 Department of Internal Medicine and Medical Specialties, A. Bianchi Bonomi Hemophilia and Thrombosis Center, Fondazione IRCCS Ca’ Granda – Ospedale Maggiore Policlinico, University of Milan, Via Pace 9, Milan 20122, Italy; 7 Department of Obstetrics and Gynaecology, Jena University Hospital, Bach Street 18, Jena 07743, Germany; 8 Independent information specialist, 3104 Apple Hill Drive, Ottawa, ON K1T 3Z2, Canada; 9 Department of Vascular Medicine, Academic Medical Center, Meibergdreef 9, Amsterdam F4-276, 1105 AZ, The Netherlands; 10 Department of Medicine, McMaster University Room HSC 3 W11, 1280 Main Street West, Hamilton, ON L8S 4 K1, Canada; 11 Clinical Trial Centre, University of Leipzig, Haertelstr 16-18, Leipzig 04107, Germany; 12 Laboratorio di Biostatistica, Centro di Ricerche Cliniche per le Malattie Rare Aldo e Cele Daccò, IRCCS - Istituto di Ricerche Farmacologiche Mario Negri, Villa Camozzi - via G. Camozzi 3, Ranica BG 24020, Italy; 13 Academic Medical Center, University of Amsterdam, Meibergdreef 9, Amsterdam 1105 AZ, The Netherlands; 14 Turku University and Satakunta Central Hospital, Helsinki University Hospital, Sairaalantie 3, 28500 Pori, Finland

**Keywords:** Pregnancy, Placenta-mediated pregnancy complications, Low-molecular-weight heparin, Meta-analysis, Individual patient data meta-analysis, Pre-eclampsia, Small-for-gestational age, Placental abruption, Pregnancy loss, Systematic review

## Abstract

**Background:**

Placenta-mediated pregnancy complications include pre-eclampsia, late pregnancy loss, placental abruption, and the small-for-gestational age newborn. They are leading causes of maternal, fetal, and neonatal morbidity and mortality in developed nations. Women who have experienced these complications are at an elevated risk of recurrence in subsequent pregnancies. However, despite decades of research no effective strategies to prevent recurrence have been identified, until recently. We completed a pooled summary-based meta-analysis that strongly suggests that low-molecular-weight heparin reduces the risk of recurrent placenta-mediated complications. The proposed individual patient data meta-analysis builds on this successful collaboration. The project is called AFFIRM, **A**n individual patient data meta-analysis o**F** low-molecular-weight heparin **F**or prevention of placenta-med**I**ated p**R**egnancy coMplications.

**Methods/Design:**

We conducted a systematic review to identify randomized controlled trials with a low-molecular-weight heparin intervention for the prevention of recurrent placenta-mediated pregnancy complications. Investigators and statisticians representing eight trials met to discuss the outcomes and analysis plan for an individual patient data meta-analysis. An additional trial has since been added for a total of nine eligible trials. The primary analyses from the original trials will be replicated for quality assurance prior to recoding the data from each trial and combining it into a common dataset for analysis. Using the anonymized combined data we will conduct logistic regression and subgroup analyses aimed at identifying which women with previous pregnancy complications benefit most from treatment with low-molecular-weight heparin during pregnancy.

**Discussion:**

The goal of the proposed individual patient data meta-analysis is a thorough estimation of treatment effects in patients with prior individual placenta-mediated pregnancy complications and exploration of which complications are specifically prevented by low-molecular-weight heparin.

**Systematic review registration:**

PROSPERO (International Prospective Registry of Systematic Reviews) 23 December 2013, CRD42013006249

## Background

Placenta-mediated pregnancy complications include pre-eclampsia (PE), late pregnancy loss, placental abruption and the small-for-gestational age (SGA) newborn. We completed a pooled summary-based meta-analysis that strongly suggests that low-molecular-weight heparin (LMWH) reduces the risk of placenta-mediated complications in subsequent pregnancies [1].

A successful pregnancy requires the development of adequate placental circulation. It has been hypothesized that thrombosis in the placental bed is at least partially responsible for placenta-mediated pregnancy complications [2-4]. It has also been suggested that these complications are the result of abnormal placental development with underdeveloped placental vasculature or placental inflammation [5,6]. These complications represent an important health problem because they are common, affecting more than one in six pregnancies [7], and often have a devastating outcome for the affected women, their unborn children, their families, and society. Specifically, PE (characterized by a new onset of elevated blood pressure and proteinuria during pregnancy) is one of the most common causes of maternal mortality in the developed world [8-11]. SGA newborns often suffer longterm effects including developmental delay, poor school performance, and a significantly lower likelihood of academic and professional success [12-14]. Fetal loss is a devastating event for pregnant women and their families. Placental abruption (separation of the placenta from the uterus before birth) can, in the most severe cases, lead to maternal hemorrhage with the risk of transfusion and both maternal and fetal death.

The risk of recurrent placenta-mediated pregnancy complications in subsequent pregnancies is substantial. For example, women with prior severe PE will have a 25 to 65% risk of recurrent PE, a 3% risk of placental abruption, and a 10% risk of SGA (<10^th^ percentile) [15,16]. These complications may be multiple (for example both PE and SGA) and not isolated to the placenta-mediated complication experienced in a prior pregnancy [15,17]. There are no highly effective preventative strategies that can be used in subsequent pregnancies. Aspirin offers small relative risk reductions in patients with prior PE and SGA, however, it may be more effective at reducing risk (approximately a 40% reduction) if started early in the pregnancy (before 16 weeks) [18,19]. There are no proven preventative strategies for the other complications. It has been postulated that anticoagulants might prevent placenta-mediated pregnancy complications by reducing placental thrombosis and/or affecting maternal coagulation activation or inflammation. Recent randomized controlled trials (RCTs) conducted to determine if LMWH can prevent recurrent placenta-mediated pregnancy complications suggest an important treatment effect [20-24], but this finding has not been universal [25].

Although it appears that LMWH is a promising therapy in the prevention of placenta-mediated pregnancy complications, there are disadvantages to the premature adoption of this intervention without sufficient evidence of benefit. If LMWH is used universally for all women with prior placenta-mediated pregnancy complications, we may be intervening unnecessarily and exposing women to a risk of undesirable and potentially fatal, albeit rare, side effects (major bleeding, heparin-induced thrombocytopenia, osteoporotic fractures, withholding of epidural analgesia due to fear of causing epidural hematoma, and paralysis) [26,27]. Less serious side effects including skin reactions, minor bleeding, and transient elevations in liver enzymes are more commonly experienced [28,29]. Therapy is also associated with cost and inconvenience since the drug is expensive and is administered by injection either once or twice a day. Therefore, it is necessary to answer the question as to who benefits from LMWH prophylaxis during pregnancy and to determine the nature and magnitude of these benefits more precisely. The individual patient data meta-analysis (IPDMA) has the potential to answer these important questions and determine the risk/benefit ratio of therapy for various subgroups of women.

The composite outcome, including all placenta-mediated pregnancy complications, that is used in many RCTs is heterogeneous and not all individual outcomes can be considered equally serious in terms of potential consequences for the mother and newborn. For example, late term pre-eclampsia is clinically less worrisome since the symptoms tend to be less severe and generally resolve with delivery. Conversely, women who develop pre-eclampsia earlier in the pregnancy have more serious clinical consequences including a greater risk of maternal and neonatal death. Our pooled summary meta-analysis suggests that LMWH may prevent severe pre-eclampsia and early pre-eclampsia with less of an effect on late onset pre-eclampsia [1]. Confirmation of these findings is extremely important for clinicians treating these women and has direct relevance for clinical practice worldwide.

There are many challenges associated with recruiting pregnant women to RCTs with a drug intervention including: the biases of clinicians either for or against the therapy (based on insufficient evidence of benefit and lack of knowledge about potential risk); the concerns of the pregnant woman and her family about the health and safety of the mother and baby; and the demands during pregnancy of attending additional appointments and investigations associated solely with study participation [1]. Furthermore, the pharmaceutical industry often excludes pregnant women from trials due to liability concerns. As a result, there is a dearth of RCTs evaluating LMWH in this population compared to other patient groups (such as oncology or orthopedic surgery). Those RCTs that do exist are all academically driven and may not have the same financial and human resources that are available to trials that are sponsored by the pharmaceutical industry. Therefore, meta-analysis is an essential tool that allows for greater statistical power by pooling the existing small RCTs evaluating LMWH for the prevention of placenta-mediated pregnancy complications.

Our recent pooled summary-based meta-analysis of six RCTs (Table 1) included 848 pregnant women with a history of pre-eclampsia, a SGA neonate (<10^th^ percentile), placental abruption, or late pregnancy loss (more than 12 weeks gestation) in a previous pregnancy [1]. The primary finding was that 67 out of 358 (18.7%) women taking LMWH during pregnancy had recurrent severe placenta-mediated pregnancy complications, as compared with 127 out of 296 (42.9%) women with no LMWH (relative risk reduction 48% (95% CI 14 to 68%; (I^2^ 69%). However, since the meta-analysis results apply to a heterogeneous group of women with a mixture of placenta-mediated pregnancy complications of varying prior severity and the primary outcome for the meta-analysis was a composite of all placenta-mediated complications (also of varying severity), it is not clear which subgroups of women derive the most benefit from LMWH (which outcomes are reduced and which severity of outcomes are impacted). Before recommendations for clinical practice can be advocated, it is necessary to conduct more detailed analyses of the existing data to determine potential benefits for subgroups of women, to adjust for important baseline characteristics of participants, and to explore other treatment-related reasons for the reported heterogeneity (for example specific LMWH drug (dalteparin, nadroparin or enoxaparin), LMWH dose, gestational age when drug was initiated, and co-interventions such as concomitant ASA use).

**Table 1:** Previously identified trials that meet the inclusion criteria for AFFIRM

**Study name & first author**	**Year**	**Country & sample size**	**Participants**	**Intervention & control**	**Outcomes**	**Commitment to participate in IPDMA**
TIPPS* [30] Rodger	2013	Canada, Multinational N = 292	Thrombophilia + previous high risk criteria	Dalteparin 5000 IU to 20 wks then 10,000 IU to 36 wks vs no Dalteparin	PE, SB, abruption, SGA <10^th^ percentile	Yes
FRUIT [20] de Vries	2012	Netherlands, Multinational N = 139	Prior early onset PE (n = 107) and/or SGA <10^th^ percentile (n = 94)	Dalteparin 5000 IU + ASA vs ASA	PE prior to 34 weeks GA	Yes
HAPPY [25] Martinelli	2012	Italy, Multi-center N = 135	Prior PE (n = 52), prior loss >15 weeks (n = 49), prior SGA <10^th^ percentile (n = 28) or prior abruption (n = 5)	Nadroparin 3800 IU vsno Nadroparin	PE, Loss >15 weeks GA, SGA <10^th^percentile and/or abruption	Yes
NOH-PE [21] Gris	2011	France, Single center N = 224	Prior severe PE (n = 224)	Enoxaparin 4000 IU + ASA vs ASA	PE, SB, abruption, SGA <5^th^ percentile	Yes
NOH-AP [24] Gris	2010	France, Single center N = 160	Prior abruption (n = 160; 70 with PE)	Enoxaparin 4000 IU+/−ASA vs +/−ASA	PE, SB, abruption, SGA <5^th^percentile	Yes
Rey [23]	2009	Canada, Multi-center N = 116	Prior early PE (n = 60), prior abruption (n = 16), prior SGA <5^th^ percentile (n = 21), loss >12 weeks (n = 17)	Dalteparin 5000 IU+/−ASA vs +/−ASA	PE, SB, abruption, SGA <5^th^ percentile	Yes
Mello [22]	2005	Italy, Single center N = 80	Prior PE with ACE DD (n = 80)	Dalteparin 5000 IU vsno Dalteparin	PE, SGA <10^th^ percentile	Unable to contact

ASA = aspirin; GA = gestational age; IPDMA = individual patient data meta-analysis; IPDMA = individual patient data meta-analysis; PE = pre-eclampsia; RCT = randomized controlled trial; SB = stillbirth; SGA = small-for-gestational age.

Trial Names:

TIPPS = Thrombophilia In Pregnancy Prophylaxis Study *accepted for publication in the Lancet.

FRUIT = FRactionated heparin in pregnant women with a history of Utero-placental Insufficiency and Thrombophilia.

NOH-AP = Nîmes Obstetricians and HAematologist – abruptio placentae.

NOH-PE = Nîmes Obstetricians and HAematologist - pre-eclampsia.

HAPPY = Heparin in pregnant women with Adverse Pregnancy outcome to improve the rate of successful PregnancY.

IPDMA has been proposed as an advantageous methodological approach when subgroup analyses are hypothesized to be clinically relevant. Analyzing original data from individual patients makes use of a much richer dataset and has greater statistical power than conventional meta-analysis [31,32]. Furthermore, for this project, IPDMA will allow for adjustment for covariates that are known to be important in the recurrence of placenta-mediated pregnancy complications. Such an analysis will also enable us to explore clinical, methodological, and statistical heterogeneity more robustly. IPDMA is an attractive method to answer our study questions since it ‘dramatically and consistently’ has more power to detect interactions between risk groups [33].

## Methods/Design

### Research questions

The primary research question is: Which women with previous placenta-mediated pregnancy complications have a reduction in the risk of future complications when treated with LMWH during pregnancy? Secondary research questions are: Which of the placenta-mediated pregnancy complications are avoided? Are severe and/or early onset or non-severe and/or late onset complications avoided? Does LMWH cause major bleeding in women with prior placenta-mediated pregnancy complications? And, are any other side effects increased by LMWH use in women with prior placenta-mediated pregnancy complications (thrombocytopenia, osteoporotic fractures or allergic reactions)?

The proposed project is called AFFIRM (**A**n individual patient data meta-analysis o**F** low-molecular-weight heparin **F**or prevention of placenta-med**I**ated p**R**egnancy complications), PROSPERO registration number: CRD42013006249. We will synthesize individual patient data from RCTs of LMWH for the prevention of recurrent placenta-mediated pregnancy complications. The overall objective of the meta-analysis is to directly inform clinical practice and the development of clinical practice guidelines. The study is coordinated by the Clinical Epidemiology Program at the Ottawa Hospital Research Institute. Conceptually, the research approach involves four sequential phases: a systematic review, knowledge synthesis planning, data extraction and analysis, and interpretation of results and knowledge translation. The first two phases have been completed and are therefore described below in the past tense. No data have been extracted or recoded for the common dataset and no statistical analyses have been performed; these steps are outlined in the future tense.

### Systematic review

**Figure 1:**
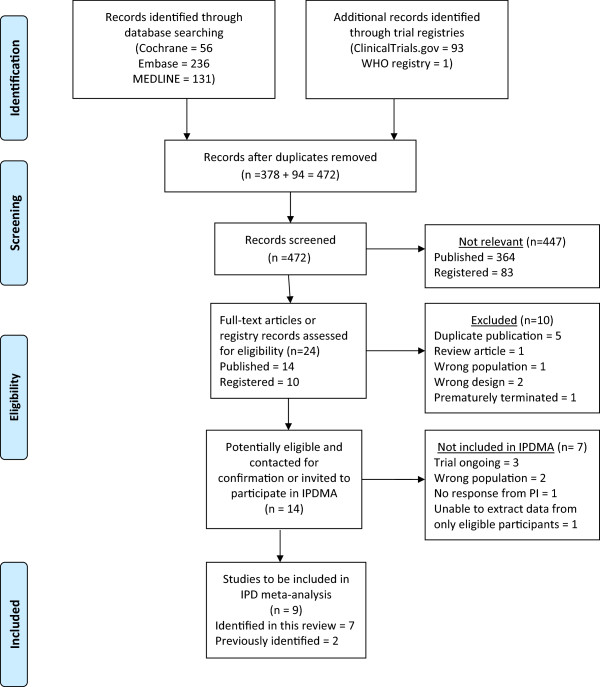
**PRISMA flow diagram of AFFIRM’s systematic review.** IPD = individual patient data; IPDMA = individual patient data meta-analysis; PRISMA = preferred reporting items for systematic reviews and meta-analyses.

Electronic search strategies were developed and tested through an iterative process by an experienced medical information specialist in consultation with the review team. The strategy was peer-reviewed prior to execution by an experienced information specialist using the Peer Review of Electronic Search Strategies (PRESS) checklist [34]. The following search was conducted in May 2013: using the OVID platform, we searched Ovid MEDLINE™, Ovid MEDLINE™ In-Process & Other Non-Indexed Citations, and EmbaseClassic + Embase (strategy included as Additional file 1). We also searched the Cochrane Library on Wiley (including CENTRAL, Cochrane Database of Systematic Reviews, DARE, and HTA). ClinicalTrials.gov and the WHO International Clinical Trials Registry were searched to identify relevant in-process and completed trials. Strategies utilized a combination of controlled vocabulary (such as ‘hypertension, pregnancy-induced’, ‘placental insufficiency’, ‘heparin, low-molecular-weight’) and keywords (pre-eclampsia, abruption, and LMWH). Vocabulary and syntax were adjusted across databases. Animal studies were excluded but there were no language or date restrictions on any of the searches. We sought additional references through hand-searching the bibliographies of relevant items. Search results are summarized in a preferred reporting items for systematic reviews and meta-analyses (PRISMA) diagram (Figure 1) and details of potentially eligible trials are provided in Tables 2 and 3.

**Table 2:** Potentially eligible published trials identified by AFFIRM’s systematic review

**Study name & first author**	**Year**	**Country & sample size**	**Participants**	**Intervention & control**	**Relevant outcomes**	**Comment re: inclusion in IPDMA**
ETHIG II *Schleussner [35]	Abstract 2013	Germany N = 449	Recurrent pregnancy loss	Dalteparin 5000 IU + vitamins vs multivitamins	Intact pregnancy at 24 wks GA; PE; IUGR <5^th^ percentile; abruption	Yes
Giancotti [36]	2012	Italy N = 167 (pregnant)	Recurrent pregnancy loss	Enoxaparin 40 mg vs Enoxaparin 40 mg + ASA vs ASA	Live births	Not eligible (All losses <12 weeks GA)
Salman [37]	Abstract 2012	Egypt N = 150	Recurrent pregnancy loss	Tinzaparin 4500 IU vs folic acid	Continuation of pregnancy after 20 weeks	Not eligible (All women with early losses)
HABENOX [38] Visser	2011	Finland, Sweden, Netherlands N = 207	Women with recurrent early or late miscarriage	Enoxaparin 40 mg vs Enoxaparin 40 mg + ASA vs ASA	Live birth rate; PE; IUGR <2 SD; abruption	Yes
SPIN [39] Clark	2010	UK, New Zealand N = 294	Recurrent pregnancy loss	Enoxaparin 40 mg + ASA vsno drug	Pregnancy loss	GA of past losses not available centrally
ALIFE [40] Kaandorp	2010	Netherlands N = 299 (pregnant)	Recurrent pregnancy loss	Nadroparin 2850 IU + ASA vs ASA vs placebo	Pregnancy loss, SGA <10^th^ percentile; PE; HELLP; abruption	Yes
HepASA [41] Laskin	2009	Canada N = 88 Terminated at interim analysis	Recurrent pregnancy loss	Dalteparin 5000 IU + ASA vs ASA	Live births	Unable to contact

ASA = aspirin; GA = gestational age; HELLP = HELLP syndrome (hemolysis, elevated liver enzymes, low platelet count); IPDMA = individual patient data meta-analysis; IUGR = intrauterine growth restriction; PE = pre-eclampsia; SB = stillbirth; SGA = small-for-gestational age.

Trial Names:

SPIN = Scottish Pregnancy Intervention Study; HepASA = Low Molecular Weight Heparin and Aspirin in the Treatment of Recurrent Pregnancy Loss.

ALIFE = Anticoagulants for Living Fetuses.

HABENOX = Low Molecular Weight Heparin and/or Aspirin in Prevention of Habitual Abortion.

ETHIG II = Effectiveness of Dalteparin Therapy as Intervention in Recurrent Pregnancy Loss *final results in preparation for publication.

**Table 3:** Potentially eligible registered trials identified by AFFIRM’s systematic review

**Study name & principal investigator**	**Identified through**	**Participants**	**Intervention & control**	**Outcomes**	**Comment re: inclusion in IPDMA**
EPPI McLintock	Ongoing RCT (New Zealand) ANZCTR registry ACTRN12609000699268	Prior PE or SGA	Enoxaparin 40 mg vs standard care	PE, SGA	Recruitment ongoing
HEPEPE Haddad	Ongoing RCT (France) Clinicaltrials.gov NCT00986765	Prior severe pre-eclampsia	Enoxaparin 4000 IU + ASA vs ASA	PE; IUGR; abruption; perinatal death	Recruitment ongoing
HOPPE Llurba	Ongoing RCT (Spain) Clinicaltrials.gov NCT01388322	Prior severe PE, SGA, loss, or abruption	Enoxaparin 40 mg or 80 mg (weight-based) vsno intervention	PE; IUGR; abruption; fetal death	Recruitment ongoing

ASA = aspirin; IPDMA = individual patient data meta-analysis; IUGR = intrauterine growth restriction; PE = pre-eclampsia; SGA = small-for-gestational age.

Trial Names:

EPPI = Enoxaparin for the Prevention of Preeclampsia and Intrauterine growth restriction.

HEPEPE = Prevention of Maternal and Perinatal Complications by Enoxaparin in Women With Previous Severe Preeclampsia (original title is French).

HOPPE = Low Weight Heparin prOphylaxis for Placental Mediated Complications of PrEgnancy.

### Inclusion criteria

RCTs with an LMWH intervention for the prevention of recurrent placenta-mediated pregnancy complications were eligible. The study population of interest included currently pregnant women with prior pregnancies complicated by one or more of the following: PE, placental abruption, SGA newborn (<10^th^ percentile), pregnancy loss after 16 weeks gestation or two losses after 12 weeks gestation. The principal investigators of potentially eligible trials identified by the systematic review (see Tables 1, 2 and 3) were contacted via email to request additional information about the study population. Once eligibility was confirmed, investigators were invited to participate in the IPDMA and attend the AFFIRM project planning meeting. The lead investigators of the largest and most recently completed trials agreed to contribute individual patient data to this collaboration. Data from two small trials [22,41] were not included because the investigators did not respond; in one of these trials only a small proportion of the total study population would have been eligible to contribute data to AFFIRM [41]. Some of the women in the Scottish Pregnancy Intervention Study (SPIN) trial would have been eligible for inclusion in AFFIRM, however, the trial database does not include sufficient detail about the timing of previous pregnancy losses to determine the eligibility of individual participants [39].

### Knowledge synthesis planning

A crucial step in the success of the project was the development of the knowledge synthesis and knowledge translation plans. A full-day review team meeting was held in Amsterdam on 4 July 2013. The purpose was to allow for extensive discussion and consensus-reaching on important study variables and outcomes and to consider strategies for merging the existing datasets in a centralized database. Participants included the principal investigators of the included RCTs and statisticians with in-depth knowledge of the trial data. The principal investigators are all practising clinicians (obstetricians and hematologists) who are also knowledge users in this clinical area.

### Outcome measures

The detailed definitions for the IPDMA outcomes were agreed upon by investigator consensus at the face-to-face meeting. The definitions and diagnostic criteria for each outcome variable are documented in a data dictionary and the research protocol. These definitions, which have been reviewed by all investigators, allow standardization across studies and decrease the potential for bias.

AFFIRM’s primary outcome is a composite outcome including four pregnancy complications: early-onset or severe pre-eclampsia, birth of a small-for-gestational age newborn with a birth weight <5^th^ percentile, placental abruption, and late pregnancy loss. To qualify as a primary outcome event, the pregnancy complication must satisfy one or more predefined criteria. Early onset pre-eclampsia is diagnosed at less than 34 weeks’ gestation. Severe pre-eclampsia is characterized by at least one criterion indicative of severe disease; these are, a systolic blood pressure ≥ 160 mm Hg or diastolic blood pressure ≥110 mm Hg, proteinuria > 0.5 g/24 hours, elevated liver enzymes (more than two times the local upper range of normal), platelets < 100 × 10^9^/L, pulmonary edema, seizures (eclampsia), headache or other neurological manifestations (stroke, intracranial hemorrhage, cerebral edema, hyperreflexia, and visual impairment), coagulopathy, oliguria (<30 ml/hr) or HELLP syndrome (hemolysis, elevated liver enzymes, low platelet count). Birth of a small-for-gestational age newborn with a birth weight <5^th^ percentile is determined using local gender and gestational age specific birth weight charts. The placental abruption outcome requires a clinical diagnosis of placental abruption leading to delivery. A late pregnancy loss occurs at or after 20 weeks of gestation and cannot be explained by other factors, including fetal chromosomal abnormalities, maternal infection, cervical insufficiency or incompetence, or an intentional termination of the pregnancy.

Nineteen secondary outcomes have been defined for AFFIRM, including the four individual components of the primary outcome: severe or early-onset pre-eclampsia, birth of a small-for-gestational age newborn <5^th^ percentile, placental abruption and late pregnancy loss, all as outlined above. Pre-eclampsia (non-severe) is characterized by a systolic blood pressure ≥140 mm Hg or diastolic blood pressure ≥90 mm Hg and proteinuria >0.3 g/24 hours. A diagnosis of HELLP syndrome required 3 criteria, hemolysis [lactate dehydrogenase (LDH) > 600 IU/L or serum bilirubin >1.2 mg/dl] an abnormal elevation of liver enzymes (more than two times the local upper range of normal), and platelets <100 × 10^9^/L. Preterm delivery <34 weeks and < 37 weeks are pre-specified outcomes. A perinatal loss is any fetal or neonatal death at over 20 weeks gestational age and less than or equal to 28 days post-partum and neonatal mortality is considered any neonatal death after birth and less than or equal to 28 days post-partum. Birth of a small-for-gestational age newborn <10^th^ percentile is determined based on local gender and gestational age specific birth weight charts.

Adverse maternal outcomes include thrombocytopenia, defined as a platelet count <75,000 × 10^9^/L, and bleeding outcomes at various time points. Antepartum major bleeding is defined using the criteria proposed by the International Society on Thrombosis and Haemostasis (ISTH) [42]. That is, clinical or radiological evidence of bleeding with at least one of the following criteria: associated with a fall in hemoglobin of 2 g/dL (1.24 mmol/L) or more; or a requirement for transfusion of two or more units of red blood cells or whole blood; or symptomatic bleeding occurring in a critical site: intracranial, intraspinal, intraocular, pericardial, intra-articular, intramuscular with compartment syndrome, or retroperitoneal, or was considered to have contributed to maternal death. Peripartum major bleeding is hemorrhage occurring after the onset of labour or start of surgical delivery and within 24 hours postpartum that meets at least one of the following: necessitating a surgical procedure, or associated with a fall in hemoglobin of 4 g/dL (2.48 mmol/L) or more, or a requirement for transfusion of two or more units of red blood cells or whole blood, or estimated peripartum blood loss >1000 ml, or considered to have contributed to maternal death. Peripartum minor bleeding is hemorrhage occurring after the onset of labour or start of surgical delivery and within 24 hours postpartum that does not meet any criterion above and with estimated peripartum blood loss between 500 and 1000 ml. Postpartum major bleeding is clinical or radiological evidence of bleeding occurring between 24 hours and 6 weeks postpartum and meeting at least one of the following ISTH criteria: associated with a fall in hemoglobin of 2 g/dL (1.24 mmol/L) or more, or a requirement for transfusion of two or more units of red blood cells or whole blood, or symptomatic bleeding occurring in a critical site: intracranial, intraspinal, intraocular, pericardial, intra-articular, intramuscular with compartment syndrome, or retroperitoneal, or considered to have contributed to maternal death.

An allergic reaction to LMWH is a reaction following the administration of LMWH that results in anaphylaxis or a rash requiring discontinuation of the allocated LMWH. Heparin-induced thrombocytopenia (HIT) is defined as a clinical diagnosis of HIT and a minimum of a positive PF4 HIT ELISA assay. The venous thromboembolism outcome includes deep vein thrombosis (DVT) and/or pulmonary embolism. The criteria for diagnosis of DVT are venography demonstrating a constant intraluminal filling defect in the deep veins above the trifurcation of the popliteal vein or compression ultrasound revealing a non-compressibility of a venous segment above the trifurcation of the popliteal vein. Diagnosis of distal, below the knee DVT, is by either venography or compression ultrasound. Diagnostic criteria for pulmonary embolism are pulmonary angiography demonstrating a constant intraluminal filling defect or a cutoff of a vessel more than 2.5 mm in diameter, or ventilation/perfusion (V/Q scan) indicating high-probability, or pulmonary embolism found at autopsy.

### Extraction and recoding of individual patient data

The definitions for each variable to be included in AFFIRM’s common dataset are documented in a data dictionary to allow standardization across studies and decrease the potential for misclassification and bias. A template for the common dataset has been developed in Microsoft Excel and will be provided to the principal investigator of each included trial. Recoded anonymized individual patient data from each of the trials will populate the Excel template. The recoded datasets for each of the individual trials will be saved on an IronKey™ USB flash drive and sent by courier to the coordinating center in Ottawa.

The AFFIRM common dataset will include individual patient data in 10 pre-defined categories: administrative and demographic data, thrombophilia, maternal medical history, pregnancy history, current pregnancy and delivery, infant data, pre-eclampsia outcome, other outcome events, intervention and treatment during pregnancy, and adverse events.

### Data synthesis, validation and analysis

Once the individual participant data from the primary studies have been merged in the common dataset, descriptive analyses will be conducted to identify data outliers, missing data, and unexpected inconsistencies. The project coordinator will prepare data clarification reports and will communicate with the principal investigators or their delegates to resolve these queries. Next, we plan to conduct preliminary analyses aimed at replicating the findings of the individual published studies, to validate the centralized database and data importation. Once the IPDMA team is satisfied with the merged dataset, the database will be locked and the planned analyses for the IPDMA synthesis will be conducted.

The individual patient data will be analyzed in a similar manner to an RCT, however, the analysis will account for clustering at the study level. The primary analysis will include all women who are eligible for AFFIRM and will examine the risk of the primary composite outcome in the treatment (LMWH) and control arms based on intention-to-treat. Secondary univariate analyses will be done for each of the pregnancy complications included in the composite outcome. On-treatment sensitivity analyses will be conducted for the primary and secondary outcomes.

### Subgroup analyses

We have planned several subgroup analyses; these were selected because they are clinically plausible and there is evidence that they may be relevant. If certain subgroups are found to be small (≤5 subjects) we will merge subgroups as appropriate.

Women will be analyzed in subgroups according to the previous pregnancy complications that were experienced. Prior pre-eclampsia subgroups are any pre-eclampsia, severe pre-eclampsia, early-onset pre-eclampsia, and severe or early onset pre-eclampsia. Subgroups according to prior SGA are SGA <10^th^ percentile, SGA <5^th^ percentile, SGA <3^rd^ percentile, prior pre-eclampsia and SGA <10^th^ percentile, prior pre-eclampsia and SGA <5^th^ percentile, prior pre-eclampsia and SGA <3^rd^ percentile. Subgroups of women with prior placental abruption are any placental abruption, placental abruption leading to delivery <37 weeks’ gestation, placental abruption leading to delivery < 34 weeks’ gestation, and placental abruption with pre-eclampsia. Participants will be grouped for analysis according to the gestational age of prior pregnancy loss: >12 weeks’ gestation, >16 weeks’ gestation, and >20 weeks’ gestation. Demographic subgroups are according to maternal age (<35 years or ≥35 years) and ethnic group (Caucasian, Black, Asian or other).

Women will be grouped according to personal characteristics and risk factors. For thrombophilia the subgroups are women with weak thrombophilia (Factor V Leiden [FVL] or prothrombin gene mutation [PGM]); moderate thrombophilia (protein C deficiency, protein S deficiency); strong thrombophilia (antithrombin deficiency, antiphospholipid antibodies, combined thrombophilia ≥1 type, homozygous FVL or PGM); or no thrombophilia. Participants will be grouped according to personal history of venous thromboembolism (VTE), family history of VTE, and no VTE history

Quality assessment will be conducted for all eligible studies using the tool for assessing risk of bias from the Cochrane Handbook for reviews of interventions [43] and reported on a study level. These assessments will also be used to inform subgroup analyses and sensitivity analyses to explore whether these biases may have affected the IPDMA analysis. We plan to examine the randomization integrity once the data from the original trials have been combined. We will endeavour to compare the original randomization lists with actual randomization to test the integrity of the allocation concealment. We will also compare the baseline characteristics of participants who have been randomized to the LMWH and no LMWH groups at the study level and aggregate level to see if there are imbalances between the groups that may suggest a lack of integrity in randomization processes.

### Knowledge translation

Once the results of the analyses are available, they will be circulated to all investigators and collaborators and a teleconference will be scheduled to discuss the findings and their interpretation. Regardless of the IPDMA results, they will be disseminated. Dr Shannon Bates is the principal knowledge user for this project. She will provide input throughout the project and will be a leader for the knowledge translation phase of the study. The principal investigators of the identified eligible RCTs (Drs Rey, Martinelli, de Vries, Gris, Rodger, Middeldorp, Schleussner, and Kaaja) are all experienced researchers and also practicing physicians who are knowledge users. Furthermore, these team members are all involved in leadership roles in their institutions and countries, including practice guideline development, and have the potential to considerably influence the international community of healthcare providers in a variety of settings.

The strategies for knowledge translation will rely heavily on the input from all involved knowledge users and will take into consideration the suitability of proposed media and/or approach for different practice settings and international contexts. Traditional methods, such as publication in a peer-reviewed journal, geared towards either a generalist or specialist audience, will be employed. Results will also be presented at international meetings; it is anticipated that knowledge users (clinicians) in hematology, obstetrics, and family medicine will be targeted. In addition, patient advocacy and education groups (such as the Pre-eclampsia Foundation, the North American Thrombosis Forum, and Thrombosis Canada) will be provided with the results in a language and format suitable to a non-medical audience.

## Discussion

This IPDMA will permit the investigators to explore which women within the heterogeneous group of patients with placenta-mediated complications benefit and which women do not benefit from low-molecular-weight heparin injections throughout pregnancy.

### Ethics, privacy and security

The subjects in each of the RCTs all provided informed consent to participate in the original trial. We will not be seeking individual consent for the secondary use of the data for the following reasons: the objectives of the IPDMA are consistent with the original trials, there are no risks or benefits associated with this analysis, no identifying information will be transferred, and it would be logistically time consuming and, in some cases, impossible to contact the women who participated. In order to ensure patient confidentiality any identifying information will be removed from the original dataset before it is transferred. The IronKey™ flash drive includes numerous security features including hardware-based encryption, a random password generator, two-factor authentication, and a self-destruct mechanism which make it extremely unlikely that the dataset can be accessed by anyone other than the intended recipient. Once the data are merged in Ottawa in the common database, they will be stored on the research institute’s network which has multiple security features and regular backup procedures in place.

### Limitations and challenges

One relevant potential drawback of IPDMA is biased pooling of data. Bias can be introduced when eligible studies are missed, when authors do not provide their data for the analysis, when the outcomes are different across studies, and when outcome and covariate data are missing from included studies [31]. Our recently completed pooled summary meta-analysis was a successful collaboration of five principal investigators [1]. In addition to the team members from these five trials, the principal investigators of four additional trials have committed to provide data for the AFFIRM meta-analysis. These are the largest and most robust trials completed in this area.

The multinational research team has representation from Canada, the Netherlands, France, Italy, Germany, and Finland. Almost all review team members attended the face-to-face IPDMA planning meeting. To protect against the misclassification of outcomes, the AFFIRM review team discussed each outcome at this meeting until consensus on detailed definitions and diagnostic criteria was reached. Definitions for all variables to be included in the IPDMA common dataset are documented in a data dictionary that was reviewed, revised according to team feedback, and finalized. Despite this, we recognize that challenges will be encountered due to variability in how the variables were originally defined and collected in each of the nine trials. In some cases it will be necessary to consult the original clinical records to obtain complete information for the IPDMA which will be a labor-intensive process. Another challenge is the diversity in language of the original datasets (English, French, Dutch, Italian, and German) that will necessitate translation when the data are recoded. Attention to detail, careful documentation, and excellent communication will be instrumental to the successful completion of this IPDMA.

## Abbreviations

ASA: Acetylsalicylic acid; ACCP: American college of chest physicians; DARE: Database of abstracts of reviews of effects; GA: Gestational age; HELLP: HELLP syndrome (hemolysis, elevated liver enzymes, low platelet count); HIT: Heparin-induced thrombocytopenia; HTA: Health technology assessment database; IPDMA: Individual patient data meta-analysis; ISTH: International society on thrombosis and hemostasis; IUGR: Intrauterine growth restriction; LMWH: Low-molecular-weight heparin; PE: Pre-eclampsia; RCT: Randomized controlled trial; SB: Stillbirth; SGA: Small-for-gestational age.

## Competing interests

Dr Marc Rodger received grant funding of more than $10,000 from Pfizer and Leo Pharma and has served on advisory boards for Sanofi Aventis but not been paid. Dr Johanna de Vries received grant funding for a two-year investigator period between 2000 and 2001 on behalf of the FRUIT-RCT by Pfizer, formerly Pharmacia. Sponsorship was obtained from Pfizer for the AFFIRM investigators’ meeting in 2013. Grant funding was obtained from Pfizer in December 2013 for a single year, to be used from January 2014 to January 2015. Dr Évelyne Rey received travel grants from Leo Pharma for the 4^th^ International Symposium on Women’s Health Issues in Thrombosis and Hemostasis, February 4-6 2011, Berlin, Germany. She also received consultant honorariums from Leo Pharma for the information booklet ‘Anticoagulation pendant la grossesse’, 2010 to 2011, and for CME presentations, 2009 to 2010. Dr Jean-Christophe Gris holds board membership for Sanofi, LFB, and Stago. He is also a consultant for Sanofi, Stago, Leo Pharma, and LFB. He has received grants from Sanofi, Stago, Leo Pharma, LFB, and Baxter Healthcare Corporation. He has received payment for lectures including service on speakers bureaus forSanofi, Stago, Leo Pharma, LFB, Bristol-Myers Squibb Pfizer, Bayer, and Boehringer Ingelheim. Dr. Shannon Bates received an honoraria from Leo Pharma and Pfizer, Canada for various presentations. Dr Saskia Middeldorp: GSK supported the ALIFE trial with a grant (until 2010). GSK currently supports the Highlow trial (has been taken over by Aspen in 2014). She has also received consulting fees and lecture honoraria from Bayer, Boehringer Ingelheim, Bristol-Myers Squibb, Pfizer, Daiichi-Sankyo and research support from GSK, Bristol-Meyers Squibb/Pfizer and Sanquin. Dr Ekkehard Schleussner: Pfizer, Germany and Merck, Germany supported the ETHIG II trial (until 2013). He also received an honoraria from Pfizer, Ferring, Bayer-Jenapharm for various presentations.

## Authors’ contributions

MR, lead IPDMA investigator; conceived of the study concept; wrote the first draft of the protocol and first draft of the manuscript; developed the IPDMA variable definitions. RM, contributed to study design, particularly data analysis (coordinating statistician); reviewed and approved of the final manuscript. TR, contributed to study design, particularly data analysis (lead statistician); critical revision of the manuscript and approval of the final manuscript. ER, JDV, MVH, ES and DP, contributed to study design; developing detailed definitions for study outcomes and eligibility; provided input on IPDMA variable definitions; critical revision of the manuscript and approval of the final manuscript. JCG, DB, AP and PDJ, contributed to study design; developing detailed definitions for study outcomes and eligibility; reviewed and approved of the final manuscript. CA, IM and SM, contributed to study design; developing detailed definitions for study outcomes and eligibility; critical revision of the manuscript and approval of the final manuscript. NL, project coordinator: AFFIRM; assisted in writing the first draft of the protocol and first draft of the manuscript; developed first draft of the data dictionary and template for the IPDMA common dataset. SB, contributed to study design, particularly knowledge translation planning; critical revision of the manuscript and approval of the final manuscript. RK, approved the methodology for the study; reviewed and approved of the final manuscript. BS, designed and conducted the electronic search strategy for the systematic review; reviewed and approved of the final manuscript. All authors read and approved the final manuscript.

## Supplementary Material

Additional file 1 Search strategy. Click here for file

## References

[B1] Rodger MA Carrier M Le Gal G Martinelli I Perna A Rey E De Vries JI Gris JC Meta-analysis of low-molecular-weight heparin to prevent recurrent placenta-mediated pregnancy complications Blood 2014 123 822 828 10.1182/blood-2013-01-478958 24357725

[B2] van der Molen EF Verbruggen B Novakova I Eskes TK Monnens LA Blom HJ Hyperhomocysteinemia and other thrombotic risk factors in women with placental vasculopathy BJOG 2000 107 785 791 10.1111/j.1471-0528.2000.tb13341.x 10847236

[B3] Weiner Z Younis JS Blumenfeld Z Shalev E Assessment of uterine placental circulation in thrombophilic women Semin Thromb Hemost 2003 29 213 218 10.1055/s-2003-38837 12709925

[B4] Arias F Romero R Joist H Kraus FT Thrombophilia: a mechanism of disease in women with adverse pregnancy outcome and thrombotic lesions in the placenta J Matern Fetal Med 1998 7 277 286 9848693 10.1002/(SICI)1520-6661(199811/12)7:6<277::AID-MFM5>3.0.CO;2-3

[B5] Sood R Kalloway S Mast AE Hillard CJ Weiler H Fetomaternal cross talk in the placental vascular bed: control of coagulation by trophoblast cells Blood 2006 107 3173 3180 10.1182/blood-2005-10-4111 16380449 PMC1895751

[B6] Steegers EA Von DP Duvekot JJ Pijnenborg R Pre-eclampsia Lancet 2010 376 631 644 10.1016/S0140-6736(10)60279-6 20598363

[B7] Berg CJ Atrash HK Koonin LM Tucker M Pregnancy-related mortality in the United States, 1987–1990 Obstet Gynecol 1996 88 161 167 10.1016/0029-7844(96)00135-4 8692494

[B8] Khan KS Wojdyla D Say L Gulmezoglu AM Van Look PF WHO analysis of causes of maternal death: a systematic review Lancet 2006 367 1066 1074 10.1016/S0140-6736(06)68397-9 16581405

[B9] Department of Health WO, Scottish Department of Health Why mothers die. Report on confidential enquiries into maternal deaths in the United Kingdom 1994–1996 1998 London: Her Majesty’s Stationary Office

[B10] Atrash HK Koonin LM Lawson HW Franks AL Smith JC Maternal mortality in the United States, 1979–1986 Obstet Gynecol 1990 76 1055 1060 2234713

[B11] Rusen ID Liston R Wen SW Bartholomew S Special Report on Maternal Mortality and Severe Morbidity in Canada. Enhanced Surveillance. The Path to Prevention 2004 Canada: Minister of Public Works and Government Services

[B12] Strauss RS Adult functional outcome of those born small for gestational age: twenty-six-year follow-up of the 1970 British Birth Cohort JAMA 2000 283 625 632 10.1001/jama.283.5.625 10665702

[B13] Strauss RS Dietz WH Effects of intrauterine growth retardation in premature infants on early childhood growth J Pediatr 1997 130 95 102 10.1016/S0022-3476(97)70316-0 9003857

[B14] Strauss RS Dietz WH Growth and development of term children born with low birth weight: effects of genetic and environmental factors J Pediatr 1998 133 67 72 10.1016/S0022-3476(98)70180-5 9672513

[B15] Van Rijn BB Hoeks LB Bots ML Franx A Bruinse HW Outcomes of subsequent pregnancy after first pregnancy with early-onset preeclampsia Am J Obstet Gynecol 2006 195 723 728 10.1016/j.ajog.2006.06.044 16949403

[B16] Sibai BM Mercer B Sarinoglu C Severe preeclampsia in the second trimester: recurrence risk and long-term prognosis Am J Obstet Gynecol 1991 165 1408 1412 10.1016/S0002-9378(12)90773-5 1957870

[B17] Hnat MD Sibai BM Caritis S Hauth J Lindheimer MD MacPherson C VanDorsten JP Landon M Miodovnik M Paul R Meis P Thurnau G Dombrowski M Perinatal outcome in women with recurrent preeclampsia compared with women who develop preeclampsia as nulliparas Am J Obstet Gynecol 2002 186 422 426 10.1067/mob.2002.120280 11904601

[B18] Askie LM Duley L Henderson-Smart DJ Stewart LA Antiplatelet agents for prevention of pre-eclampsia: a meta-analysis of individual patient data Lancet 2007 369 1791 1798 10.1016/S0140-6736(07)60712-0 17512048

[B19] Bujold E Roberge S Lacasse Y Bureau M Audibert F Marcoux S Forest JC Giguere Y Prevention of preeclampsia and intrauterine growth restriction with aspirin started in early pregnancy: a meta-analysis Obstet Gynecol 2010 116 402 414 10.1097/AOG.0b013e3181e9322a 20664402

[B20] De Vries JIP Van Pampus MG Hague WM Bezemer PD Joosten JH Low-molecular-weight heparin added to aspirin in the prevention of recurrent early-onset pre-eclampsia in women with inheritable thrombophilia: the FRUIT-RCT J Thromb Haemost 2012 10 64 72 10.1111/j.1538-7836.2011.04553.x 22118560

[B21] Gris JC Chauleur C Molinari N Mares P Fabbro-Peray P Quere I Lefrant JY Haddad B Dauzat M Addition of enoxaparin to aspirin for the secondary prevention of placental vascular complications in women with severe pre-eclampsia. The pilot randomised controlled NOH-PE trial Thromb Haemost 2011 106 1053 1061 10.1160/TH11-05-0340 21946915

[B22] Mello G Parretti E Fatini C Riviello C Gensini F Marchionni M Scarselli GF Gensini GF Abbate R Low-molecular-weight heparin lowers the recurrence rate of preeclampsia and restores the physiological vascular changes in angiotensin-converting enzyme DD women Hypertension 2005 45 86 91 10.1161/01.HYP.0000149950.05182.a3 15557391

[B23] Rey E Garneau P David M Gauthier R Leduc L Michon N Morin F Demers C Kahn SR Magee LA Rodger M Dalteparin for the prevention of recurrence of placental-mediated complications of pregnancy in women without thrombophilia: a pilot randomized controlled trial J Thromb Haemost 2009 7 58 64 10.1111/j.1538-7836.2008.03230.x 19036070

[B24] Gris JC Chauleur C Faillie JL Baer G Mares P Fabbro-Peray P Quere I Lefrant JY Haddad B Dauzat M Enoxaparin for the secondary prevention of placental vascular complications in women with abruptio placentae. The pilot randomised controlled NOH-AP trial Thromb Haemost 2010 104 771 779 10.1160/TH10-03-0167 20694277

[B25] Martinelli I Ruggenenti P Cetin I Pardi G Perna A Vergani P Acaia B Facchinetti F La Sala GB Bozzo M Rampello S Marozio L Diadei O Gherardi G Carminati S Remuzzi G Mannucci PM Heparin in pregnant women with previous placenta-mediated pregnancy complications: a prospective, randomized, multicenter, controlled clinical trial Blood 2012 119 3269 3275 10.1182/blood-2011-11-391383 22289887 PMC3321853

[B26] Huhle G Geberth M Hoffmann U Heene DL Harenberg J Management of heparin-associated thrombocytopenia in pregnancy with subcutaneous r-hirudin Gynecol Obstet Invest 2000 49 67 69 10.1159/000010216 10629377

[B27] Greer IA Nelson-Piercy C Low-molecular-weight heparins for thromboprophylaxis and treatment of venous thromboembolism in pregnancy: a systematic review of safety and efficacy Blood 2005 106 401 407 10.1182/blood-2005-02-0626 15811953

[B28] Bank I Libourel EJ Middeldorp S van der Meer J Buller HR High rate of skin complications due to low-molecular-weight heparins in pregnant women J Thromb Haemost 2003 1 859 861 10.1046/j.1538-7836.2003.t01-7-00115.x 12871432

[B29] Rodger MA Thrombophilia and placenta-mediated pregnancy complications: from the bench to bedside to policy Thromb Res 2008 123 S96 S100 10.1016/S0049-3848(09)70021-0 19217464

[B30] Rodger MA Kahn SR Cranney A Hodsman A Kovacs MJ Clement AM Lazo-Langner A Hague WM Long-term dalteparin in pregnancy not associated with a decrease in bone mineral density: substudy of a randomized controlled trial J Thromb Haemost 2007 5 1600 1606 10.1111/j.1538-7836.2007.02634.x 17663731

[B31] Sud S Douketis J ACP Journal Club. The devil is in the details…or not? A primer on individual patient data meta-analysis Ann Intern Med 2009 151 JC1 JC3 10.7326/0003-4819-151-1-200907070-00122 19620147

[B32] Clarke MJ Individual patient data meta-analyses Best Pract Res Clin Obstet Gynaecol 2005 19 47 55 10.1016/j.bpobgyn.2004.10.011 15749065

[B33] Lambert PC Sutton AJ Abrams KR Jones DR A comparison of summary patient-level covariates in meta-regression with individual patient data meta-analysis J ClinEpidemiol 2002 55 86 94 10.1016/s0895-4356(01)00414-0 11781126

[B34] Sampson M McGowan J Cogo E Grimshaw J Moher D Lefebvre C An evidence-based practice guideline for the peer review of electronic search strategies J Clin Epidemiol 2009 62 944 952 10.1016/j.jclinepi.2008.10.012 19230612

[B35] Schleussner E Kamin G Seeliger G Rogenhofer N Toth B for the ETHIG Investigator Group Low-molecular-weight heparin in recurrent pregnancy loss - Results of the ETHIG II study [abstract] Thromb Res 2013 131 S73

[B36] Giancotti A La TR Spagnuolo A D'Ambrosio V Cerekja A Piazze J Chistolini A Efficacy of three different antithrombotic regimens on pregnancy outcome in pregnant women affected by recurrent pregnancy loss J Matern Fetal Neonatal Med 2012 25 1191 1194 10.3109/14767058.2011.600366 21988715

[B37] Salman SA Shaaban OM Zahran KM Fathalla MM Anan MA Low molecular weight heaprin (LMWH) for treatment of recurrent miscarriage negatively tested for antiphospholipid antibodies: a randomized controlled trial [abstract] Fertil Steril 2012 98 S191 10.1177/1076029616665167 27572887

[B38] Visser J Ulander VM Helmerhorst FM Lampinen K Morin-Papunen L Bloemenkamp KW Kaaja RJ Thromboprophylaxis for recurrent miscarriage in women with or without thrombophilia. HABENOX: a randomised multicentre trial Thromb Haemost 2011 105 295 301 21103659 10.1160/TH10-05-0334

[B39] Clark P Walker ID Langhorne P Crichton L Thomson A Greaves M Whyte S Greer IA SPIN: the Scottish Pregnancy Intervention Study: a multicentrerandomised controlled trial of low molecular weight heparin and low dose aspirin in women with recurrent miscarriage Blood 2010 115 4162 4167 10.1182/blood-2010-01-267252 20237316

[B40] Kaandorp SP Goddijn M van der Post JA Hutten BA Verhoeve HR Hamulyak K Mol BW Folkeringa N Nahuis M Papatsonis DN Papatsonis DN Buller HR van der Veen F Middeldorp S Aspirin plus heparin or aspirin alone in women with recurrent miscarriage N Engl J Med 2010 362 1586 1596 10.1056/NEJMoa1000641 20335572

[B41] Laskin CA Spitzer KA Clark CA Crowther MR Ginsberg JS Hawker GA Kingdom JC Barrett J Gent M Low molecular weight heparin and aspirin for recurrent pregnancy loss: results from the randomized, controlled HepASA Trial J Rheumatol 2009 36 279 287 10.3899/jrheum.080763 19208560

[B42] Schulman S Kearon C Definition of major bleeding in clinical investigations of antihemostatic medicinal products in non-surgical patients J Thromb Haemost 2005 3 692 694 10.1111/j.1538-7836.2005.01204.x 15842354

[B43] Higgins JPT Green S Cochrane Handbook for Systematic Reviews of Interventions Version 5.1.0 [updated March 2011] 2011 The Cochrane Collaboration [http://www.cochrane-handbook.org]

