# Supplementary material for: Low-molecular-weight heparin for prevention of placenta-mediated pregnancy complications: protocol for a systematic review and individual patient data meta-analysis (AFFIRM)
Source: Syst Rev. 2014 Jun 26;3:69. doi: 10.1186/2046-4053-3-69 (PMC4094595; doi:10.1186/2046-4053-3-69)
Supplement: Additional file 1 — Search strategy. [file 2046-4053-3-69-S1.pdf]

Appendix 1. Search Strategy

LWMH & Placenta-Mediated Pregnancy Complications
OVID 2013 May 5

Database: Embase Classic+Embase <1947 to 2013 May 03>, Ovid MEDLINE(R) In-Process & Other Non-Indexed Citations and Ovid MEDLINE(R) <1946 to Present>
Search Strategy:
----
1 exp Hypertension, Pregnancy-Induced/ (35044)
2 (pregnan* adj5 hypertensi*).tw. (20960)
3 ((gestational or maternal) adj5 hypertens*).tw. (7212)
4 PIH.tw. (3198)
5 (((high* or rais* or elevat* or heighten* or increas*) adj3 (blood pressure or diastolic pressure or systolic pressure or pulse pressure)) and pregnan*).tw. (4438)
6 (((high* or rais* or elevat* or heighten* or increas*) adj3 (BP or DBP or SBP)) and pregnan*).tw. (756)
7 (eclamp* or pre-eclamp* or preeclamp*).tw. (51722)
8 (EPH adj1 (Complex* or Gestos* or Toxemi* or Toxaemi* or Syndrome*)).tw. (1163)
9 ((Edema or oedema) and Proteinuria and Hypertension and Gestosis).tw. (118)
10 ((pregnan* or gestational or gravidum or gravidarum) adj5 (toxemi* or toxaemi*)).tw. (9496)
11 HELLP.tw. (4291)
12 (Hemolysis and Elevated Liver and Lowered Platelet*).tw. (1)
13 Infant, Small for Gestational Age/ (11668)
14 "small for gestational age".tw. (12359)
15 SGA.tw. (9803)
16 Fetal Growth Retardation/ (30362)
17 ((intrauterine or intra-uterine) adj2 growth restrict*).tw. (8013)
18 ((intrauterine or intra-uterine) adj2 growth retard*).tw. (12905)
19 ((fetal or foetal or fetus* or foetus*) adj2 growth restrict*).tw. (5530)
20 ((fetal or foetal or fetus* or foetus*) adj2 growth retard*).tw. (4811)
21 IUGR.tw. (8668)
22 exp Fetal Death/ or Stillbirth/ (57294)
23 (stillbirth* or stillborn*).tw. (23406)
24 ((fetal or foetal or fetus* or foetus* or prenatal* or pre-natal* or perinatal* or peri-natal* or antepartum or ante-partum or antenatal* or ante-natal*) adj3 (loss* or death*)).tw. (35608)
25 exp Abortion, Spontaneous/ (53089)
26 (abort* adj3 (spontaneous* or habitual* or frequen* or recur* or tubal)).tw. (26088)
27 (miscarriage* or miscarry or miscarries or miscarried or miscarrying).tw. (20236)
28 ((second trimester* or 2nd trimester* or third trimester* or 3rd trimester* or late pregnan* or advanced pregnan* or late intrauterine or late intra-uterine) adj3 (loss* or death*)).tw. (1044)
29 Placental Insufficiency/ (3986)
30 ((placent* or uteroplacent* or utero-placenta*) adj3 (insufficien* or incompeten* or failure*)).tw. (4744)
31 Abruptio Placentae/ (6398)
32 (placent* adj1 (abruptio* or ablation* or detachment* or separation* or solutio* or apoplexia*)).tw. (6756)
33 abruption*.tw. (3994)
34 (placent* and vascular and thrombos*).tw. (449)
35 (("placenta-mediated pregnancy" or "placental-mediated pregnancy") adj3 (complicat* or

problem* or difficult* or disorder*)).tw. (49)
36 "Pregnancy Complications, Hematologic"/ (81830)
37 exp Placenta/de (4123)
38 or/1-37 (345669)
39 exp Heparin, Low-Molecular-Weight/ (47677)
40 LMWH.tw. (8183)
41 ((low molecular weight or LMW) adj1 heparin).tw. (17403)
42 Heparin, Low-Molecular-Weight.rn. (6540)
43 (Dalteparin* or FR-860 or Fragmin or Fragmine or Kabi-2165 or "K 2165'" or K2165 or Tedelparin or low liquemin).tw. (3901)
44 dalteparin.rn. (713)
45 (Enoxaparin* or Clexan* or EMT-966 or EMT-967 or HSDB 7846 or Klexane or Lovenox or PK10169 or PK 10169 or "PK-10,169" or RP 54563 or UNII-8NZ41MIK1O).tw. (9385)
46 enoxaparin.rn. (16124)
47 (nadroparin* or CY 216 or CY 216d or CY216 or CY216d or Fraxiparin* or LMF CY-216 or Nadroparin Calcium or Nadroparine or Nadrohep or Fraxodi or Seleparin* or Tedegliparin*).tw. (2607)
48 nadroparin.rn. (3999)
49 (tinzaparin* or Innohep or UNII-7UQ7X4Y489).tw. (1145)
50 tinzaparin.rn. (229)
51 (bemiparin* or hibor or phivor or ardeparin* or UNII-N3927D01PB).tw. (285)
52 (certoparin* or Alphaparin* or Alpha-parin* or Mono-Embolex or Monoembolex).tw. (357)
53 (Reviparin* or Clivarin* or LU 47311 or LU47311 or lomorin).tw. (492)
54 reviparin.rn. (83)
55 (parnaparin* or parvoparin* or fluxum or lohepa or lowhepa or minidalton or op 2123 or CB-01-05-MMX).tw. (237)
56 Parnaparin.rn. (33)
57 (semuloparin* or mulsevo or visamerin or AVE 5026 or AVE5026 or UNII-4QW4AN84NQ).tw. (101)
58 semuloparin.rn. (6)
59 sevuparin*.tw. (3)
60 sevuparin.rn. (0)
61 (ardeparin* or normifio or normiflo or rd heparin or wy 90493 or wy90493).tw. (199)
62 ardeparin.rn. (22)
63 (adomiparin* or "m 118" or m118).tw. (153)
64 adomiparin.rn. (0)
65 ("cy 222" or cy222).tw. (233)
66 cy 222.rn. (168)
67 (danaproid or "kb 101" or kb101 or lomoparan or lomoparin or mucoglucuronan or org 10172 or org10172 or orgaran).tw. (1507)
68 danaproid.rn. (402)
69 deligoparin*.tw. (3)
70 deligoparin.rn. (0)
71 ((heparin adj1 dihydergot) or (dihydroergotamine adj1 heparin) or Embolex or (heparin adj1 DHE)).tw. (458)
72 heparin-dihydergot.rn. (73)
73 idrabiotaparinux.tw. (60)
74 idrabiotaparinux.rn. (81)
75 idraparinux.tw. (287)
76 idraparinux.rn. (640)

77 livaraparin calcium.tw. (0)
78 livaraparin calcium.rn. (0)
79 minolteparin*.tw. (0)
80 minolteparin.rn. (0)
81 rd 11885.tw. (13)
82 rd 11885.rn. (0)
83 tafoxiparin*.tw. (3)
84 tafoxiparin.rn. (1)
85 tedelparin*.tw. (23)
86 tedelparin.rn. (0)
87 or/39-86 (54271)
88 38 and 87 (2563)
89 exp Animals/ not (Humans/ and exp Animals/) (8585219)
90 88 not 89 (2543)
91 randomized controlled trial.pt. (347918)
92 randomized controlled trials as topic/ or random allocation/ or double-blind method/ or single-blind method/ or placebos/ (696235)
93 (random* or RCT$1 or placebo*).tw. (1618914)
94 ((singl* or doubl* or trebl* or tripl*) and (mask* or blind* or dumm*)).tw. (312130)
95 trial.ti. (267139)
96 or/91-95 (2087101)
97 90 and 96 (370)
98 meta analysis.pt. (39487)
99 exp meta-analysis as topic/ (19716)
100 (meta-analy* or metanaly* or metaanaly* or met analy* or integrative research or integrative review* or integrative overview* or research integration or research overview* or collaborative review*).tw. (122093)
101 (systematic review* or systematic overview* or evidence-based review* or evidence-based overview* or (evidence adj3 (review* or overview*)) or meta-review* or meta-overview* or "review of reviews" or technology assessment* or HTA or HTAs).tw. (152082)
102 exp Technology assessment, biomedical/ (20394)
103 health technology assessment winchester england.jn. (1195)
104 (evidence report technology assessment or evidence report technology assessment summary).jn. (398)
105 "cochrane database of systematic reviews".jn. (12972)
106 or/98-105 (284631)
107 90 and 106 (102)
108 limit 90 to systematic reviews [Limit not valid in Embase; records were retained] (1936)
109 97 or 107 or 108 (2026)
110 109 use prmz (133)
111 maternal hypertension/ (8849)
112 (pregnan* adj5 hypertensi*).tw. (20960)
113 (gestational adj5 hypertens*).tw. (4492)
114 (maternal adj5 hypertens*).tw. (3166)
115 PIH.tw. (3198)
116 (((high* or rais* or elevat* or heighten* or increas*) adj3 (blood pressure or diastolic pressure or systolic pressure or pulse pressure)) and pregnan*).tw. (4438)
117 (((high* or rais* or elevat* or heighten* or increas*) adj3 (BP or DBP or SBP)) and pregnan*).tw.

(756)
118 exp pregnancy toxemia/ (66763)
119 (eclamp* or pre-eclamp* or preeclamp*).tw. (51722)
120 (EPH adj1 (Complex* or Gestos* or Toxemi* or Toxaemi* or Syndrome*)).tw. (1163)
121 ((Edema or oedema) and Proteinuria and Hypertension and Gestosis).tw. (118)
122 ((pregnan* or gestational* or gravidum or gravidarum) adj5 (toxemi* or toxaemi* or toxicos*)).tw. (10458)
123 HELLP syndrome/ (4441)
124 HELLP.tw. (4291)
125 (Hemolysis and Elevated Liver and Lowered Platelet*).tw. (1)
126 exp intrauterine growth retardation/ (37399)
127 "small for gestational age".tw. (12359)
128 SGA.tw. (9803)
129 ((intrauterine or intra-uterine) adj2 growth restrict*).tw. (8013)
130 ((intrauterine or intra-uterine) adj2 growth retard*).tw. (12905)
131 ((fetal or foetal or fetus* or foetus*) adj2 growth restrict*).tw. (5530)
132 ((fetal or foetal or fetus* or foetus*) adj2 growth retard*).tw. (4811)
133 IUGR.tw. (8668)
134 exp fetus death/ (32616)
135 (stillbirth* or stillborn*).tw. (23406)
136 ((fetal or foetal or fetus* or foetus* or prenatal* or pre-natal* or perinatal* or peri-natal* or antepartum or ante-partum or antenatal* or ante-natal*) adj3 (loss* or death*)).tw. (35608)
137 spontaneous abortion/ (38858)
138 (abort* adj3 (spontaneous* or habitual* or frequen* or recur* or tubal)).tw. (26088)
139 (miscarriage* or miscarry or miscarries or miscarried or miscarrying).tw. (20236)
140 ((second trimester* or 2nd trimester* or third trimester* or 3rd trimester* or late pregnan* or advanced pregnan* or late intrauterine or late intra-uterine) adj3 (loss* or death*)).tw. (1044)
141 placenta insufficiency/ (2667)
142 ((placent* or uteroplacenta* or utero-placenta*) adj3 (insufficien* or incompeten* or failure*)).tw. (4744)
143 solutio placentae/ (4671)
144 (placent* adj1 (abruptio* or ablation* or detachment* or separation* or solutio*)).tw. (6753)
145 abruption*.tw. (3994)
146 (placent* and vascular and thrombos*).tw. (449)
147 (("placenta-mediated pregnancy" or "placental-mediated pregnancy") adj3 (complicat* or problem* or difficult* or disorder*)).tw. (49)
148 (pregnan* and (hematolog* adj5 (complicat* or problem* or difficult* or disorder*))).tw. (392)
149 or/111-148 (264754)
150 exp low molecular weight heparin/ (47677)
151 LMWH.tw. (8183)
152 ((low molecular weight or LMW) adj1 heparin).tw. (17403)
153 (Dalteparin* or FR-860 or FR860 or Fragmin or Fragmine or Kabi-2165 or K-2165 or K2165 or Tedelparin* or low liquemin).tw. (3901)
154 (Enoxaparin* or Clexan* or EMT-966 or EMT966 or EMT-967 or EMT967 or HSDB 7846 or Klexane or Lovenox or PK 10169 or PK10169 or "PK-10,169" or RP 54563 or UNII-8NZ41MIK1O).tw. (9385)
155 679809-58-6.rn. (5397)
156 (nadroparin* or CY 216 or CY 216d or CY216 or CY216d or Fraxiparin* or LMF CY-216 or Nadroparin Calcium or Nadroparine or Nadrohep or Fraxodi or Seleparin* or Tedegliparin*).tw. (2607)

157 (tinzaparin* or Innohep or logiparin* or lhn1 or UNII-7UQ7X4Y489).tw. (1304)
158 (bemiparin* or hibor or phivor or ardeparin* or UNII-N3927D01PB).tw. (285)
159 (certoparin* or Alphaparin* or Alpha-parin* or Mono-Embolex).tw. (283)
160 (reviparin* or clivarin* or LU47311 or "LU 47311" or lomorin).tw. (492)
161 (parnaparin* or parvoparin* or fluxum or lohepa or lowhepa or minidalton or op 2123 or "CB-01-05-MMX").tw. (237)
162 (semuloparin* or mulsevo or visamerin or AVE 5026 or AVE5026 or UNII-4QW4AN84NQ).tw. (101)
163 sevuparin*.tw. (3)
164 (ardeparin* or normifio or normiflo or rd heparin or "wy 90493" or wy90493).tw. (199)
165 (adomiparin* or "m 118" or m118).tw. (153)
166 antixarin*.tw. (5)
167 (cy 222 or cy222).tw. (233)
168 (danaproid or "kb 101" or kb101 or lomoparan or lomoparin or mucoglucuronan or org 10172 or org10172 or orgaran).tw. (1507)
169 308068-55-5.rn. (0)
170 deligoparin*.tw. (3)
171 ((heparin adj1 dihydergot) or (dihydroergotamine adj1 heparin) or Embolex or (heparin adj1 DHE)).tw. (458)
172 idrabiotaparinux.tw. (60)
173 idraparinux.tw. (287)
174 162610-17-5.rn. (574)
175 livaraparin calcium.tw. (0)
176 minolteparin*.tw. (0)
177 rd 11885.tw. (13)
178 tafoxiparin*.tw. (3)
179 tedelparin*.tw. (23)
180 or/150-179 (54093)
181 149 and 180 (2088)
182 exp animals/ or exp animal experimentation/ or exp models animal/ or exp animal experiment/ or nonhuman/ or exp vertebrate/ (36963297)
183 exp humans/ or exp human experimentation/ or exp human experiment/ (27357123)
184 182 not 183 (9607791)
185 181 not 184 (2067)
186 randomized controlled trial/ (691922)
187 "randomized controlled trial (topic)"/ (29863)
188 randomization/ (138604)
189 double blind procedure/ (119069)
190 single blind procedure/ (17360)
191 placebo/ (234074)
192 (random* or RCT$1 or placebo*).tw. (1618914)
193 ((singl* or doubl* or trebl* or tripl*) and (mask* or blind* or dumm*)).tw. (312130)
194 or/186-193 (1997796)
195 185 and 194 (340)
196 "systematic review"/ (59764)
197 meta-analysis/ (110106)
198 "meta analysis (topic)"/ (7056)
199 (meta-analy* or metanaly* or metaanaly* or met analy* or integrative research or integrative review* or integrative overview* or research integration or research overview* or collaborative

review*).tw. (122093)
200 (systematic review* or systematic overview* or evidence-based review* or evidence-based overview* or (evidence adj3 (review* or overview*)) or meta-review* or meta-overview* or "review of reviews" or technology assessment* or HTA or HTAs).tw. (152082)
201 biomedical technology assessment/ (19303)
202 (cochrane or health technology assessment or evidence report).jw. (23566)
203 or/196-202 (321170)
204 185 and 203 (129)
205 195 or 204 (390)
206 205 use emczd (317)
207 110 or 206 (450) TOTAL HITS
208 remove duplicates from 207 (367) TOTAL HITS AFTER DE-DUPING
209 208 use prmz (131) UNIQUE MEDLINE HITS
210 208 use emczd (236) UNIQUE EMBASE HITS

****
